# The Abundance and Diversity of Soil Fungi in Continuously Monocropped Chrysanthemum

**DOI:** 10.1155/2013/632920

**Published:** 2013-10-23

**Authors:** Aiping Song, Shuang Zhao, Sisi Chen, Jiafu Jiang, Sumei Chen, Huiyun Li, Yu Chen, Xi Chen, Weimin Fang, Fadi Chen

**Affiliations:** College of Horticulture, Nanjing Agricultural University, Nanjing 210095, China

## Abstract

Chrysanthemum is an important ornamental plant which is increasingly being monocropped. Monocropping is known to affect both fungal abundance and species diversity. Here, quantitative PCR allied with DGGE analysis was used to show that fungi were more abundant in the rhizosphere than in the bulk soil and that the fungal populations changed during the growth cycle of the chrysanthemum. The majority of amplified fragments appeared to derive from *Fusarium* species, and *F. oxysporum* and *F. solani* proved to be the major pathogenic species which are built up by monocropping.

## 1. Introduction

Chrysanthemum (*Chrysanthemum morifolium*) is an important ornamental species, particularly in China. With the increasingly urbanized Chinese population experiencing a marked rise in its standard of living, chrysanthemum production is rising. As is also the case for many crops [1–5], long-term monocropping of chrysanthemum depresses crop productivity, in terms of both quantity and quality. One of the major causes for this decline in productivity is thought to be the changed nature of the soil fungal population and specifically the buildup of soil-borne pathogens. Soil fungi are important in the context of nutrient cycling and transport and carbon recycling [6–8]. Although some fungal species are pathogenic to plants, there are also those which are pathogenic to insect pests and so are clearly beneficial [9]. Only a small proportion of the full spectrum of soil fungi species is readily isolatable using *in vitro *culture, but species identification and quantification methods based on the polymerase chain reaction (PCR) have made a considerable impact in this field. In particular, the combination of a PCR-based assay of the variable 18S rRNA gene and amplicon separation using denaturing gradient gel electrophoresis (DGGE) has been used to derive a much more complete picture of the soil fungal community than what has been achievable in the past [10].

The physical and chemical environment in the rhizosphere is heavily influenced by the living root [11–13], which also affects the local abundance and diversity of soil microbes [14]. Soil-borne pathogens are attracted to the roots of their host species via their perception of specific molecules secreted by the plant into the soil [15, 16]. In a monocropping situation, it is this mechanism which is largely responsible for the buildup of pathogen inoculum over time.

As yet there has been little research focus on the soil microbial community associated with ornamental species. The dynamics of the bacterial component of the chrysanthemum soil microflora were described in some detail by Duineveld et al. [17, 18], but no published literature relates to the fungal component of the soil microflora. Here, we have investigated fungal abundance and diversity in soil supporting the growth of chrysanthemum using real time PCR and DGGE. The aims were to assess whether fungal abundance and diversity were affected by the growth stage of the plant and/or by continuous monocropping and to identify which fungal species are responsible for productivity decline in monocropped chrysanthemum. 

## 2. Materials and Methods

### 2.1. Soil and Plant Growth

Soil used for three years of continuously monocropped chrysanthemum was obtained from the Chrysanthemum Germplasm Resource Preserving Centre, Nanjing Agricultural University, Nanjing, China. Its pH was 6.0, and it contained 10% organic matter and ~15% moisture. Cuttings of the cultivar “Jinba” (obtained from the Chrysanthemum Germplasm Resource Preserving Centre) were first established by growing in a perlite medium for three weeks then transplanted into pots; meanwhile, the soil mentioned above was applied. The material was raised in a greenhouse maintained at 28°C during the 16 h day and at 22°C during the night; the relative humidity was kept at 70%. Eight weeks after transplantation, the photoperiod was reduced to 8 h to induce flowering.

### 2.2. Soil Sampling and Extraction of Soil Microflora DNA

Two, six, and 12 weeks after the transplanting, rhizosphere and bulk soil samples were collected and combined from 10 individually grown plants following Zhao et al. [19]. At these times, the plants were at the seedling stage, the vegetative stage, and the productive stage, respectively. All samples were taken at 9:00 a.m. to avoid any diurnal effect [20]. Each sample contained the rhizosphere soil of ten plants. Genomic DNA was extracted from the soil samples using a NucleoSpin Soil kit (MACHEREY-NAGEL, Germany).

### 2.3. Real Time PCR

The abundance of fungal species was estimated by the real time PCR analysis of 18S rDNA amplicons, as described by Fierer et al. [21] with minor modifications. Each 20 *μ*L reaction contained 10 *μ*L SYBR *Premix Ex Taq* II (Takara, Japan), 0.5 *μ*M of each of the primers (ITS1f and 5.8s, Table 1), and 2.5 ng template DNA. The amplification regime comprised a 5 min denaturation step at 95°C, followed by 40 cycles of 95°C/15 s, 53°C/30 s, and 72°C/45 s. Standard curves were generated using a ten-fold serial dilution (from 10^9^ to 10^4^ copies per *μ*L) of a plasmid containing a full length copy of the *Saccharomyces cerevisiae* 18S rRNA gene [22]. All reactions were run in three replicates with the DNA extracted from each soil sample and the technically appropriate set of standards. The data were analyzed by Student's *t*-test (with a level of significance of 0.01) using the software package SPSS 17.

### 2.4. PCR Amplification for DGGE

The components of the fungal microflora were identified using a PCR assay based on variation in the 18S rDNA gene. The forward primer employed was Fung-GC, and the reverse primer was NS1 (Table 1) [23]. A total of 25 *μ*L of PCR mixture contained 1 × *Ex Taq* PCR buffer with MgCl_2_, 100 *μ*M dNTP, 0.5 *μ*M of each of the primers, 1U *Ex Taq* DNA polymerase (Takara), and 50 ng DNA template. The amplification regime comprised a denaturing step (94°C/5 min), followed by 25 cycles of 94°C/30 s, 56°C/30 s, and 72°C/60 s, and finally an extension step of 72°C/10 min. The amplicon (expected size ~350 bp) was separated by agarose electrophoresis and visualized by EtBr staining.

### 2.5. DGGE Analysis and Sequence Analysis of Selected Fragments

The DGGE procedure employed 8% polyacrylamide gels (ratio of acrylamide to bisacrylamide: 37 : 1) formed with a denaturing gradient of 25 to 45% (where 100% represented 7 M urea, 40% v/v formamide) [26]. Electrophoresis was carried out at 60°C and 80 V for 16 h using the D-code system (Bio-Rad, USA). The gels were stained for 30 min with DuGreen nucleic acid gel stain (Fanbo Biochemicals, China), which fluoresces in the presence of UV light. Selected DNA fragments were excised from the DGGE gel and submerged overnight at 4°C in 100 *μ*L TE buffer. A PCR based on a 1 *μ*L aliquot of the gel fragment extract as template was performed under the same conditions as described above, with the Fung primer replacing Fung-GC as the forward primer (Table 1) [23]. The resulting amplicons were purified using a Biospin Gel Extraction kit (BioFlux, China) and cloned into the pMD19-T vector (Takara) for sequencing. Recovered sequences were scanned by BLAST [27] against the GenBank nucleotide sequence database.

### 2.6. Fungal Diversity Analysis

The DGGE profiles were analyzed by Quantity One 4.4.0 software (Bio-Rad) to obtain a measure of fungal diversity. Each band was considered as a single operational taxonomic unit, and a phylogeny was generated based on the UPGMA algorithm. Richness (*S*) was given by the number of distinct bands in a given profile. The diversity index *H*′ [28] was calculated from the expression −∑(*p*_*i*_)(ln⁡*p*_*i*_), where *p* was the proportion of an individual band's gradation relative to the sum of all bands' gradation and *p*_*i*_ the relative abundance of fragment *i*. The index of diversity 1/*D* [29] was calculated from the expression 1/∑(*p*_*i*_)^2^, and evenness (*E*) was given by *H*/*H*_max⁡_, where *H*_max⁡_ = ln⁡(*S*). 

### 2.7. Isolation and Identification of Pathogenic Species

Chrysanthemum seedlings were planted into soil which had been continuously monocropped to chrysanthemum for three years, and infecting fungi were recovered from diseased root, stem, and rhizome material using the conventional organizational separation [30, 31]. The identification of fungal species was carried out by applying both DNA diagnostics and morphological characterization. The former involved the PCR amplification and sequencing of the nuclear ribosomal repeat unit internal transcribed spacer (ITS) sequence, based on the ITS1F and ITS4 primers (Table 1) [24, 25]. The ITS sequences of isolated strains were scanned by BLAST [27] against the GenBank nucleotide sequence database. The morphological characterization involved the front and back cultures characters of isolates cultured on potato dextrose agar (PDA) and the form of the macro- and microconidia [32].

### 2.8. Pathogen Bioassay

Spore suspensions of putative pathogens were obtained from 14-day-old cultures on PDA by adding sterile water to the surface of the Petri dish. The suspension was subsequently filtered through four layers of cheesecloth [2], and the spore concentration was adjusted to 1 × 10^7^ CFU per mL using a haemocytometer. Plants were inoculated and scored after 28 days, following the methods given by Huang et al. [33]. 

## 3. Results

### 3.1. Abundance of Soil Fungi

**Figure 1:**
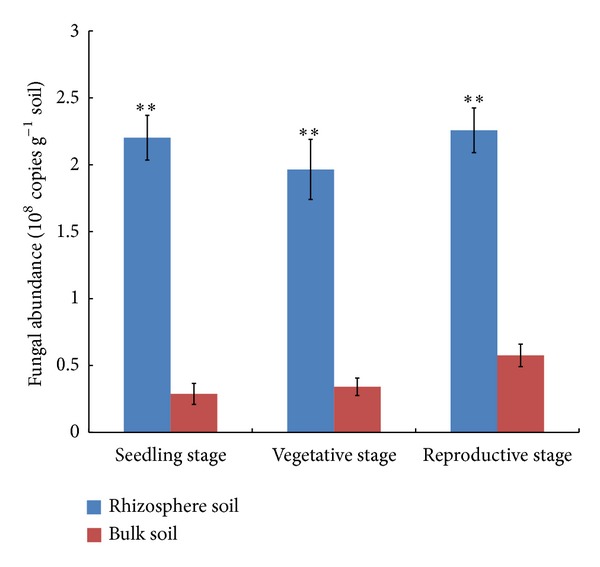
The abundance of fungi in the rhizosphere and bulk soil, as indicated by a real time PCR estimation of the copy number of an 18S rDNA fragment. Standard error bars calculated from three replicates. Significant differences based on Student's *t*-test indicated by asterisks (**P < 0.01).

**Figure 2:**
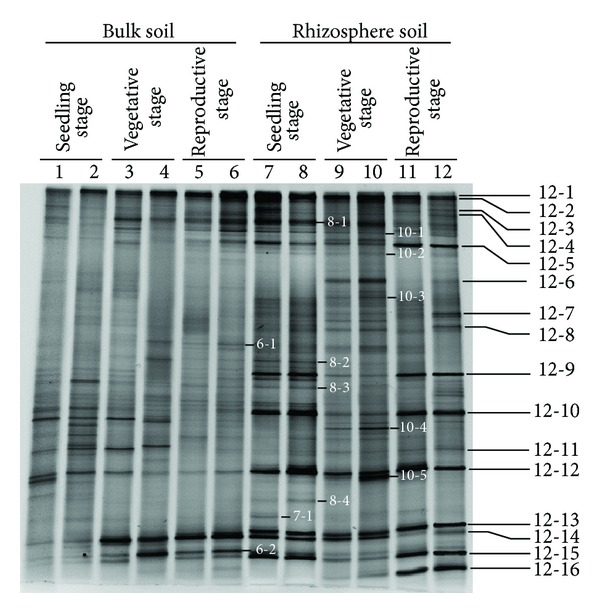
DGGE profiles of 18S rDNA fragments present in DNA extracted from bulk soil (lanes 1–6) and rhizosphere (lanes 7–12) sampled at various developmental stages of growing chrysanthemum plants. Lanes 1, 2, 7, and 8: seedling stage, lanes 3, 4, 9, and 10: vegetative stage, and lanes 5, 6, 11, and 12: reproductive stage. Fragments excised for sequencing are indicated by numbers (Table 2).

**Figure 3:**
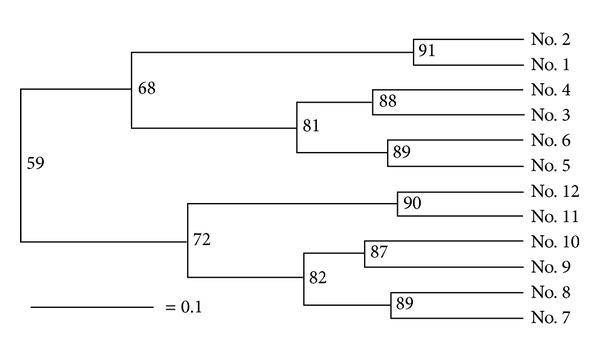
Phylogeny of the soil microflora, derived from 18S rDNA DGGE profiles. No. 1–no. 6: bulk soil samples and no. 7–no. 12: rhizosphere samples. No. 1, no. 2, no. 7, and no. 8: soil from plants at the seedling stage, no. 3, no. 4, no. 9, and no. 10: at the vegetative stage, and no. 5, no. 6, no. 11, and no. 12: at the reproductive stage.

As estimated from the output of the real time PCR, the number of fungal colony-forming units per gram of rhizosphere soil (cfu g^−1^) was 2.20 × 10^8^ at the seedling stage, 1.97 × 10^8^ at the vegetative stage, and 2.26 × 10^8^ at the reproductive stage; these levels of abundance were all significantly higher than what was present in the bulk soil (resp., 0.29, 0.34, and 0.58 × 10^8^ cfu g^−1^) (Figure 1). The DNA extracted from every soil sample tested positive when amplified using the fungal 18S rDNA primers Fung-GC and NS1, and 28 amplified fragments were taken forward for sequencing (Figure 2). The BLAST results associated with some of these, as detailed in Table 2, implied that a number of common plant pathogens were well represented: these included *Magnaporthe grisea* (rice blast),* Rhizoctonia solani* (wide host range), and the two *Fusarium *spp., *F. oxysporum *and *F. solani*. The latter two species were particularly well represented in the rhizosphere during the seedling and reproductive stages depending on the DGGE bands' profiles, while the presence of *R. solani *was detected at all three stages of chrysanthemum development, but at a lower intensity. Evidence of the presence of beneficial fungi in the rhizosphere was provided by the amplification of product from *Chaetomium globosum* (Band 12–6) (Figure 2). The DGGE profiles indicated that the complexity and abundance of soil fungi was greater in the rhizosphere samples than in the bulk soil (Figure 2). A comparison between the two profiles suggested a level of similarity of 59% based on the UPGMA algorithm, and the recovery of the same fragment from duplicate samples showed that the DNA isolation, PCR, and electrophoretic procedures had all been reliable (Figure 3). Overall, *Ascomycete *species were the most abundant (68% of all identified species, Table 2), followed by *Basidiomycetes* (21%).

### 3.2. Analysis of Fungal Diversity

Our results demonstrated that the fungi diversity in the rhizosphere soil was different from that in the bulk soil (Table 3). In the rhizosphere soil sampled from plants at the vegetative stage, *S* (35), *H*′ (3.48), and 1/*D* (29.53) were greater than in the bulk soil sampled from plants at the same stage (*S* = 20, *H*′ = 2.85, 1/*D* = 14.75). The value of *S* in the bulk soil was also lower than that in the rhizosphere soil at the seedling stage, but during the reproductive stage, *S* was higher in the bulk soil. However, the *E* parameter remained relatively constant throughout, lying in the range of 0.95–0.99. 

### 3.3. The Isolation and Bioassay of Pathogens Isolated from Diseased Chrysanthemum

**Figure 4:**
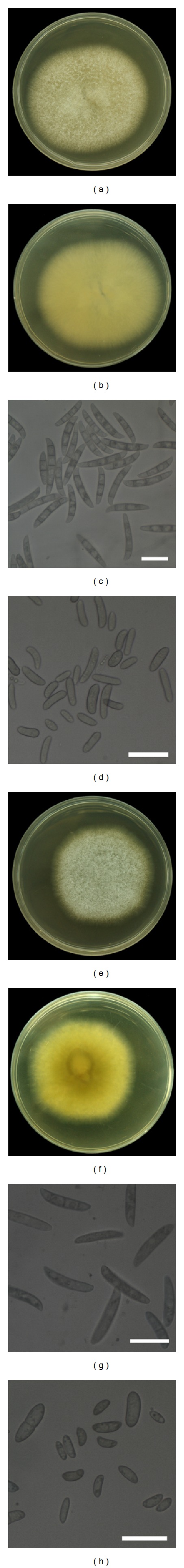
Morphology of *F. solani* isolate CFD-1 (a–d) and *F. oxysporum* isolate CFD-1 (e–h). (a, e): front culture character, (b, f): back culture character, (c, g): macroconidia, and (d, h): microconidia. Bars: 50 *μ*m.

**Figure 5:**
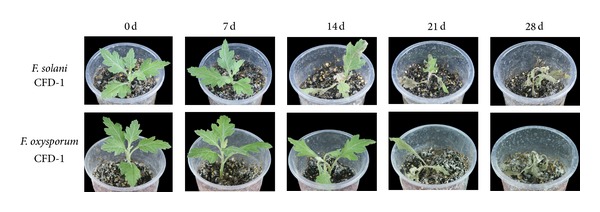
The temporal development of disease symptoms in chrysanthemum plants inoculated with either *F. solani *CFD-1 or *F. oxysporum *CFD-1.

After five days of *in vitro *culture, 15 fungal strains were isolated from various diseased plant tissues. On the basis of their ITS sequences, it was possible to identify that 11 of these 15 isolates shared 97% similarity with *F. solani* and the other four shared 98% similarity with *F. oxysporum*. One of the putative *F. solani *strains (CFD-1, see Figures 4(a)–4(d)) and one of the putative *F. oxysporum* strains (CFD-1, Figures 4(e)–4(h)) were used for a reinoculation test. The resulting wilt index and infection rate measured 28 days after inoculation (dpi) were 3.6 and 96.3% for *F. solani* CFD-1 and 3.7 and 97.9% for *F. oxysporum* CFD-1 (Table 4). The wilt index following inoculation with *F. solani *CFD-1 was zero at seven dpi, 1.2 at 14 dpi, and 1.9 at 21 dpi, while the time course development of disease following inoculation with *F. oxysporum *CFD-1 was zero at seven dpi, 0.8 at 14 dpi, and 2.1 at 21 dpi. The appearance of the plants as the disease developed is displayed in Figure 5. The pathogen reisolated from the inoculated plants was identical to the one used for the inoculation by ITS sequencing and morphology.

## 4. Discussion

Plants exert a strong influence on the structure and turnover of the rhizosphere fungal community [34–36]. There was little evidence from the current experiments that the abundance of fungi, either in the rhizosphere or in the bulk soil, was responsive to the developmental stage of the chrysanthemum plant (Figure 1). This lack of response may be related to the way in which the soil microflora had been influenced by continuous monocropping. Fungi were more abundant in the rhizosphere than in the bulk soil, presumably because carbohydrate-based exudates from the plant root encouraged the development of a localized higher microbial population size [13, 36, 37].

It has been recognized that a molecular marker-based method of characterizing the components of a complex population can be affected by biases arising from any one of the DNA extraction protocol, the choice of primers, and differential PCR amplifiability [38]. However, it has been demonstrated that a reduced number of PCR cycles and mixing replicate reactions do reduce the risk of bias [39, 40], and this was therefore the approach adopted here to maximize the probability that any differences identified were not experimental artefacts.

The diversity of the DGGE profiles and the variation in the relative abundance of specific amplicons showed that rhizosphere is a significant driver of the structure of the soil microflora community. Furthermore, the plant development stage also influenced fungi diversity significantly, a result which is inconsistent with the claim that the plant only has a minor influence on the constitution of the rhizosphere fungal community [20, 41]. The reason for this inconsistency was likely that the different soil types and sampling methods lead to the different results. 

The incidence of wilting in continuously monocropped chrysanthemum crops is most frequent at the seedling stage, followed by during the reproductive stage, but only occurs rarely during the vegetative stage (data not shown). The generally held belief is that this wilting is the consequence of the buildup of soil-borne pathogens over the previous cropping cycle(s) [2, 4]. The present investigation suggested a potential explanation. The abundance of *F. oxysporum* and *F. solani* was at its peak during the seedling stage, while during the vegetative stage it decreased at the same time as the abundance of beneficial fungi increased (Figure 2). If, as has been suggested by Yu and Matsui [42], the constitution of root exudates is developmentally regulated, then the expectation is that the fungal community will also vary qualitatively over the course of the plants' development. 

The reinoculation test showed that the isolates were indeed pathogenic. This makes it highly likely that the *Fusarium* spp. in question are responsible for the wilt affecting continuously monocropped chrysanthemum. These results may promote the prevention and early diagnosis of *Fusarium* wilt disease, which was prevalent in continuously monocropped chrysanthemum. The abundance of these fungi in the rhizosphere is encouraged by exudates produced by the chrysanthemum root. The present study has established a firm foundation for studying the interaction between the chrysanthemum plant and its pathogenic and beneficial rhizosphere fungi. 

## Figures and Tables

**Table 1:** Sequences of the primer sets used.

Primer	Sequence	Reference
ITS1f	TCCGTAGGTGAACCTGCGG	Fierer et al., 2005 [21]
5.8S	CGCTGCGTTCTTCATCG	Fierer et al., 2005 [21]
Fung	ATTCCCCGTTACCCGTTG	Möhlenhoff et al., 2001 [23]
Fung-GC	CGCCCGCCGCGCCCCGCGCCCGGCCCGCCGCCCCCGCCCCATTCCCCGTTACCCGTTG	Möhlenhoff et al., 2001 [23]
NS1	GTAGTCATATGCTTGTCTC	Möhlenhoff et al., 2001 [23]
ITS1F	CTTGGTCATTTAGAGGAAGTA	Gardes and Bruns, 1993 [24]
ITS4	TCCTCCGCTTATTGATATGC	White et al., 1990 [25]

**Table 2:** Most closely related sequences to those derived from selected 18S rDNA amplicons separated by DGGE.

Band(s)	Most closely related bacterial sequence	Identity (%)	Accession no.
12-1	*Magnaporthe grisea* strain Guy 11	98%	AF277123.1
12-2	Uncultured *Cyathus* clone F3	98%	EF640307.1
12-3	*Trichoderma koningiopsis* strain T-440	100%	JQ278020.1
12-4	*Coniochaeta ligniaria *	99%	AY198389.1
8-1	*Fusarium* sp. EF1	100%	GQ166777.1
10-1	*Myceliophthora hinnulea* strain ATCC 52474	100%	JQ067909.1
12-5	*Cordyceps sinensis *	99%	AB067700.1
10-2	*Rhizoctonia solani* isolate Q1	99%	JF499071.1
12-6	*Chaetomium globosum* isolate NK-102	98%	HQ529774.1
10-3	*Termitomyces clypeatus* isolate TB	98%	HM036344.1
12-7	*Bionectria ochroleuca* strain WY-1	97%	GU112755.1
12-8	*Cyphelium tigillare *	100%	AF241545.1
6-1	*Trechispora alnicola* isolate AFTOL-ID 665	98%	AY657012.1
8-2	*Chrysomphalina grossula *isolate AFTOL-ID 981	99%	AY752969.1
12-9	*Fusarium oxysporum* strain SP-2	99%	HM152769.1
8-3	*Fusarium *sp. MBS1	100%	FJ613599.1
12-10	*Fusarium solani *strain 421502	98%	EF397944.1
10-4	*Aspergillus ustus *isolate Li-62	99%	GU573851.1
12-11	*Acremonium sclerotigenum* strain CBS 124.42	98%	HQ232209.1
12-12	*Campanella* sp. MCA2235	98%	AY916675.1
10-3	*Crinipellis zonata *strain OKM 25450	99%	AY916691.1
8-4	Uncultured fungus isolate DGGE gel band 22	95%	JN591717.1
7-1	*Hypocrea jecorina *strain EIM-30	98%	JN831373.1
12-13	*Geomyces destructans* isolate MmyotGER-1	100%	GU999983.1
12-14	*Pythium cylindrosporum* isolate 275	99%	EU199112.1
6-2	*Pythium boreale* strain CBS 551.88	99%	EF418927.1
12-15	Uncultured soil *basidiomycete* clone F7	100%	JN656541.1
12-16	*Emericellopsis maritima* isolate AFTOL-ID 999	98%	FJ176807.1

**Table 3:** Diversity indices associated with the fungal flora present in the rhizosphere and bulk soil samples of continuously monocropped chrysanthemum.

Diversity index	Bulk soil			Rhizosphere soil		
	Seedling stage	Vegetative stage	Reproductive stage	Seedling stage	Vegetative stage	Reproductive stage
*S*	23	20	30	28	35	26
*H*′	3.1	2.85	3.26	3.23	3.48	3.15
*E*	0.99	0.95	0.96	0.97	0.98	0.97
1/*D*	21.47	14.75	21.27	23.06	29.53	21.44

*S*: richness, *H*′: Shannon-Wiener diversity index, *E*: evenness, 1/*D*: the reciprocal of Simpson's index of diversity.

**Table 4:** The pathogenicity of two *Fusarium *sp. isolates present in diseased chrysanthemum plants.

Strains	No. of plants inoculated	Infection rate (%)	Wilt index^a^
*Fusarium solani* CFD-1	160	96.3	3.6
*Fusarium oxysporum* CFD-1	144	97.9	3.7

^a^Representing wilt index at 28 dpi, 0: no wilting; 1: slight wilting on some leaves; 2: most leaves wilted; 3: leaves severely wilted but green; 4: plants wilted and dead.
